# Fluorescent sperm in a transparent worm: validation of a GFP marker to study sexual selection

**DOI:** 10.1186/1471-2148-14-148

**Published:** 2014-06-30

**Authors:** Lucas Marie-Orleach, Tim Janicke, Dita B Vizoso, Micha Eichmann, Lukas Schärer

**Affiliations:** 1 Evolutionary Biology, Zoological Institute, University of Basel, Vesalgasse 1, CH-4051 Basel, Switzerland; 2 Centre d’Écologie Fonctionnelle et Évolutive, 1919 route de Mende, FR-34293 Montpellier, France

**Keywords:** Cryptic female choice, Fertilisation, Genetic engineering, Sperm competition, Sperm displacement, Sperm labelling, Sperm tracking, Sperm storage, Transgenesis

## Abstract

**Background:**

Sexual selection has initially been thought to occur exclusively at the precopulatory stage in terms of contests among males and female mate choice, but research over the last four decades revealed that it often continues after copulation through sperm competition and cryptic female choice. However, studying these postcopulatory processes remains challenging because they occur internally and therefore are often difficult to observe. In the transparent free-living flatworm *Macrostomum lignano,* a recently established transgenic line that expresses green fluorescent protein (GFP) in all cell types, including sperm, offers a unique opportunity to non-invasively visualise and quantify the sperm of a GFP-expressing donor inside the reproductive tract of wild-type recipients *in vivo*. We here test several aspects of the reproductive performance of the transgenic individuals and the accuracy of the techniques involved in assessing the GFP-expressing worms and their sperm. We then show the usefulness of these methods in a study on sperm displacement.

**Results:**

GFP-expressing worms do not differ from wild-type worms in terms of morphology, mating rate and reproductive success. In addition, we show that the GFP signal is reliably and unequivocally expressed by all GFP-expressing individuals observed under epifluorescence illumination. However, the intensity of the GFP signal emitted by sperm of GFP expressing donors can vary (which we show to be at least in part due to sperm ageing) and the GFP marker is inherited according to Mendel’s laws in most, but not all, of the individuals. Nevertheless, we argue these two issues can be addressed with an appropriate experimental design. Finally, we demonstrate the value of the GFP-techniques by comparing the number of GFP-expressing sperm in a wild-type recipient before and after mating with a competing sperm donor, providing clear experimental evidence for sperm displacement in *M. lignano*. This result suggests that sperm donors can displace previously stored sperm and replace it with their own.

**Conclusion:**

The availability of the GFP-techniques in a transparent organism provide unique opportunities to visualise and quantify internal processes in the female reproductive tract after mating, which opens new avenues in the study of sexual selection.

## Background

Sexual selection was first defined by Darwin as the selection that “depends on the advantage which certain individuals have over others of the same sex and species solely in respect of reproduction” [1]. Sexual selection theory intends to explain, for instance, why male red deer (*Cervus elaphus*) engage in impressive battles or why peacocks (*Pavo cristatus*) display colourful features. This likely happens because individuals that outcompete rivals (e.g. via male-male competition) and/or attract mating partners (e.g. via female mate choice) gain mating opportunities that consequently lead to a higher reproductive success [2]. In addition to the competition for mating opportunities, it has been realised that sexual selection can continue after copulation. Specifically, when females mate multiply and store sperm, ejaculates from different males may compete for fertilisation (sperm competition) and females may also influence the fertilisation success of some males by preferentially using their sperm (cryptic female choice) [3-9]. Postcopulatory sexual selection has been argued to be an important evolutionary force [10,11] and to shape for example the evolution of “giant sperm” in *Drosophila hydei* that are 10 times the adult body length [12], or harmful male genitalia in seed beetles [13].

A large number of studies have inferred mechanisms behind postcopulatory sexual selection from the patterns of paternity skews. This has for instance been achieved by combining paternity analyses with behavioural manipulations (e.g. [14-17]), or with artificial insemination to control for potential differences in the number of sperm inseminated (e.g. [18-21]). However, because these approaches focus on the ultimate fitness outcome of postcopulatory sexual selection, they yield limited insights about the underlying mechanisms from which the skews in paternity result. Postcopulatory processes are indeed challenging to observe and to quantify, mainly because they often occur inside the female reproductive tract. As a consequence, one remaining enigma in sexual selection research is how selection acts on sperm once it is stored in the female reproductive tract and further progress in our understanding of postcopulatory sexual selection requires techniques that allow the observation of internal processes.

Some established methods already shed some light on the cryptic nature of these internal processes and permit to assign the relative contributions of donors to a pool of sperm inside the female reproductive tract *in situ*. To our knowledge, this can currently be achieved through the following five methods. First, when a morphological sperm trait has a nonoverlapping range between donors, this trait may be used as a marker to distinguish sperm from different donors (e.g. sperm length, [22]). Second, sperm can be experimentally radiolabelled, using either amino-acids or nucleotides containing specific radioisotopes, which can later be quantified in the recipient by scintillation counting or autoradiography (e.g. [23,24]). Third, sperm cell DNA can be labelled with a halogenated pyrimidine (such as bromodeoxyuridine, BrdU) integrated during spermatogenesis, which can later be tracked in the recipient by using immunocytochemical staining techniques (e.g. [25-27]). Fourth, in *Drosophila melanogaster*, transgenic lines have been established that express fluorescent markers in sperm, e.g. green or red fluorescent proteins, which enables the visualisation of competing ejaculate *in situ* and the quantification of sperm behaviour (e.g. [28-31]). And fifth, the competitive PCR approach allows the quantification of donor-specific genetic markers, such as microsatellites, in the sperm stored in the reproductive tract of a recipient (e.g. [32-35]). These opportunities to quantify the contributions of specific donors to a pool of sperm stored within a recipient have greatly improved our understanding of postcopulatory sexual selection, including insights on sperm transfer, sperm storage, sperm displacement, sperm dynamics and cryptic female choice [24,26,29,34]. However, all of these methods have a common limitation because they involve destructive sampling, requiring either to dissect the female reproductive tract or to fixate the entire sperm recipient and so, the sperm recipient can no longer be used for paternity analysis or further experimental manipulations. Owing to this experimental limitation, the link between sperm storage and fitness of both sexes (ultimately translating into selection on the stored sperm and/or the female that is storing the sperm) remained largely unexplored until now.

**Figure 1:**
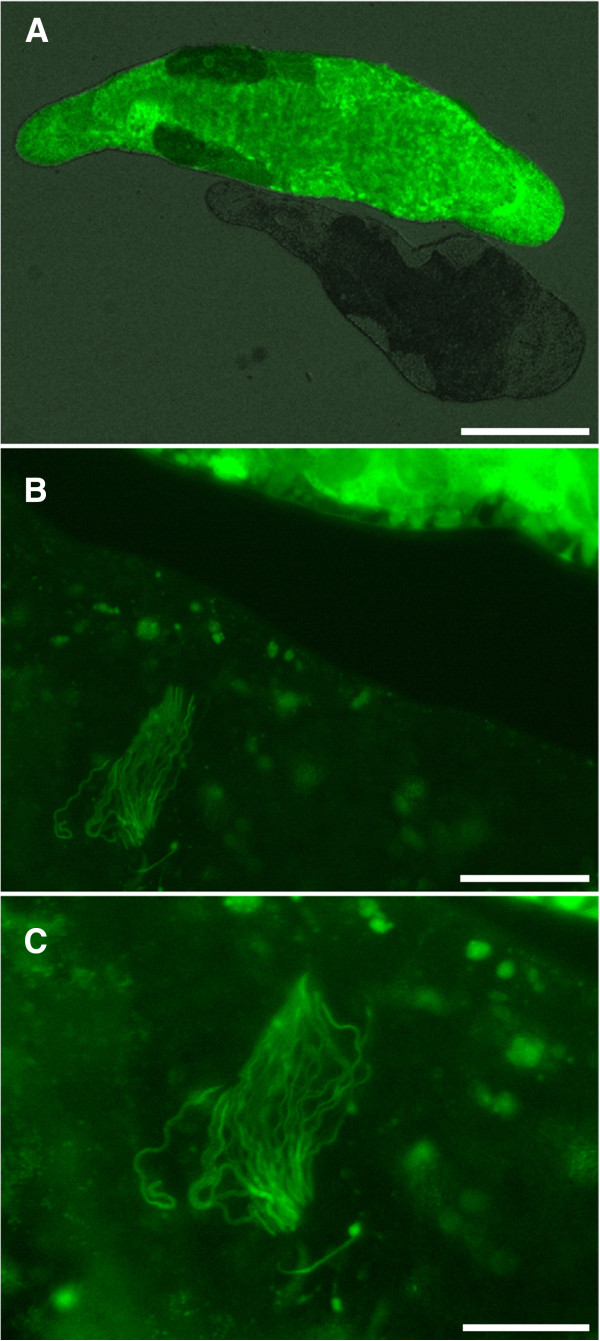
***In vivo *micrographs of a GFP-expressing individual [hereafter GFP(+)] and a wild type individual [hereafter GFP(-)]. A**, the GFP(+) worm, bright green, next to the GFP(-) worm, dark gray. **B** and **C**, increasingly magnified views of the sperm storage organ of the GFP(-) worm containing fluorescent sperm received from the GFP(+) worm. Scale bars represent 400, 60 and 40 μm in **A**, **B** and **C**, respectively.

**Figure 2:**
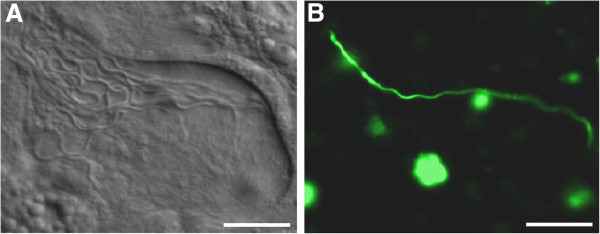
***In vivo *micrographs of the antrum of a worm mated with a GFP(+) and a GFP(-) partner. A**, total number of received sperm under bright field illumination (14 sperm cells). **B**, the single fluorescent sperm coming from the GFP(+) sperm donor, under epifluorescence illumination. Scale bar represents 20 μm.

Here we present a study system which allows us to track the sperm of a specific sperm donor *in vivo* under competitive conditions using the non-invasive visualisation of labelled sperm inside the female reproductive tract of a transparent sperm recipient. This breakthrough has become possible due to a recently established transgenic line of the free-living flatworm *Macrostomum lignano*[36], which expresses green fluorescent protein (GFP) in all cell types, including the sperm (Figure 1 and 2). This technique allows real-time observation of the interactions between sperm of a given donor with those of competitors inside the female reproductive tract of a living sperm recipient. The non-invasive nature of this approach adds unique opportunities to the previously established methods. For example it allows us to directly study the selection episode from sperm storage to fertilisation and to quantify selection operating on traits that bias sperm fertilising success. Furthermore, it allows us to repeatedly assess the contribution of a sperm donor within a pool of received sperm and therefore to study temporal patterns of sperm storage within recipients. This technique offers novel opportunities to study mechanisms of postcopulatory sexual selection and thus to obtain new insights on sexual selection in general. However, to fully evaluate the usefulness of this method we need to determine if any traits relevant for reproduction differ between GFP(+) and GFP(-) individuals, and to assess the reliability of using GFP label for identification of individuals and sperm.

We report on a series of tests that clearly establish the reliability and potential of the GFP techniques, and we discuss limitations that need to be taken into account in future experiments. Furthermore, in order to illustrate the experimental power of the described GFP technique we studied sperm displacement in *M. lignano.* For this, we assessed the number of sperm received from a GFP(+) donor twice in the same recipient, before and after mating to a second GFP(-) sperm donor. The results unambiguously demonstrate the presence of sperm displacement in *M. lignano*.

## Methods and results

### Study species and strains used

*Macrostomum lignano* (Macrostomorpha, Platyhelminthes) is a free-living flatworm from the intertidal zone of the Northern Adriatic Sea that is easily cultured under laboratory conditions, where it reaches about 1.5 mm and has a generation time of about 18 days [37]. It is an outcrossing simultaneous hermaphrodite that mates frequently, has reciprocal copulations (i.e., donates and receives sperm during a single copulation) and possesses distinct pre- and postcopulatory behaviours that can be easily observed and quantified [38,39]. Worms are transparent, allowing non-invasive observation and reliable measurements of the size of different internal structures such as testis, ovary and seminal vesicle [40,41]. The received sperm can be counted inside the sperm-storage organ (hereafter antrum) [42]. Thus, due to this ability to quantify several reproductive traits, *M. lignano* has emerged as a suitable model organism to study sexual selection.

In this study, we investigate whether transgenic GFP(+) individuals differ from GFP(-) individuals in several aspects of their reproductive performance, and thus whether the GFP-techniques can be reliably used to study sexual selection. For all the tests, we used two lines, a GFP(+) line (called HUB1; [36]) and a GFP(-) line (called DV1; [43]). As explained in Janicke et al. [43], DV1 was created via full-sib and half-sib inbreeding for 24 generations, and has since been maintained at a small population size to maintain inbreeding. More recently, the DV1 line was used to create a stable transgenic line expressing GFP, the HUB1 line [36] and so, the HUB1 and DV1 lines are expected to be almost identical genetically. Briefly, transgenesis was achieved by micro-injecting a DNA construct into a single cell stage egg, leading to stable and ubiquitous GFP-expression in all cell types, including sperm. The DNA construct contained a DNA region of a transposable element (MINOS), the promoter region of a *Macrostomum* housekeeping gene (elongation factor alpha), and the coding sequence of a GFP protein (eGFP). Details on the establishment of the HUB1 line are described in Demircan [36].

### General methods

#### *Generating same-age individuals*

To reduce among-individual variation due to age, we used same-age individuals in all experiments. For this, we transferred well-fed adult individuals into glass Petri dishes with f/2 medium [44], allowing individuals to lay eggs for 1 or 2 days, after which all adults were removed, ensuring the resulting hatchlings did not differ in age by more than 1 or 2 days.

#### *Raising conditions*

Soon after hatching, the resulting same-age hatchlings were collected and distributed in wells of 24-well tissue culture plates (TPP, Switzerland) filled with 1.5 mL of f/2 medium and fed *ad libitum* with the algae *Nitzschia curvilineata*. Then, individuals were regularly transferred (every 7 to 10 days) to new wells with fresh algae, until they reached sexual maturity.

#### *Colouring individuals*

To be able to distinguish individual worms under normal light, we coloured the worms by using the vital dye patent blue V (also called E-131, Werner Schweizer AG, Switzerland), diluted to a concentration of 0.25 mg/mL of f/2 medium. A 24 h exposure enables us to colour individuals, and has previously been shown to not affect the mating rate [39].

#### *Assessing received sperm in the female antrum*

To observe the sperm in the antrum, we followed an established protocol [42,43]. Briefly, we prepared an observation chamber where anaesthetized worms are squeezed in between two cover slips. Then, by using a microscope connected to a camera, we recorded movies of the entire antrum in which the sperm can be visualised *in vivo*. Bright field illumination permits the visualisation of the total number of sperm, whereas epifluorescence illumination permits the visualisation of the fluorescent sperm only. Subsequently, we counted the total sperm and fluorescent sperm in storage based on movies, while being blind with respect to the different treatments. Importantly, the strength of the GFP signal of the fluorescent sperm seemed constant over the span of the observation.

#### *Statistics*

All statistics analyses were carried out in JMP 10.0.1 (SAS Institute Inc., Cary, NC, U.S.A.).

### Experiment 1. Morphology of GFP(+) vs. GFP(-) individuals

#### *Experimental setup*

To test if individuals of the GFP(+) and GFP(-) lines differ in their morphology, we raised individuals in groups of either 2 or 8 individuals, and measured a suite of morphological traits. Specifically, we raised same-age individuals in pairs, i.e. 1 GFP(+) and 1 GFP(-) individual, or in octets, i.e. 4 GFP(+) and 4 GFP(-) individuals. We then took morphological measurements following the standard protocol described elsewhere [41], including body size, testis size, ovary size and seminal vesicle size. We measured both individuals in the pairs, and one randomly sampled individual of each line in the octets.

#### *Statistics*

The sample size was 19 pairs and 25 octets for all traits, except for seminal vesicle size for which the sample size was 18 pairs and 24 octets. Body size and testis size were log transformed, and seminal vesicle size was square-root transformed. To test for morphological differences between the lines, we fitted linear mixed models (LMM) independently for the 4 response variables (i.e. body size, testis size, ovary size and seminal vesicle size), and used the line [i.e. GFP(+) or GFP(-)], the social group size (i.e. pair or octet) and the line by social group size interaction as fixed effects, and the identity of the group as a random effect.

#### *Results*

**Figure 3:**
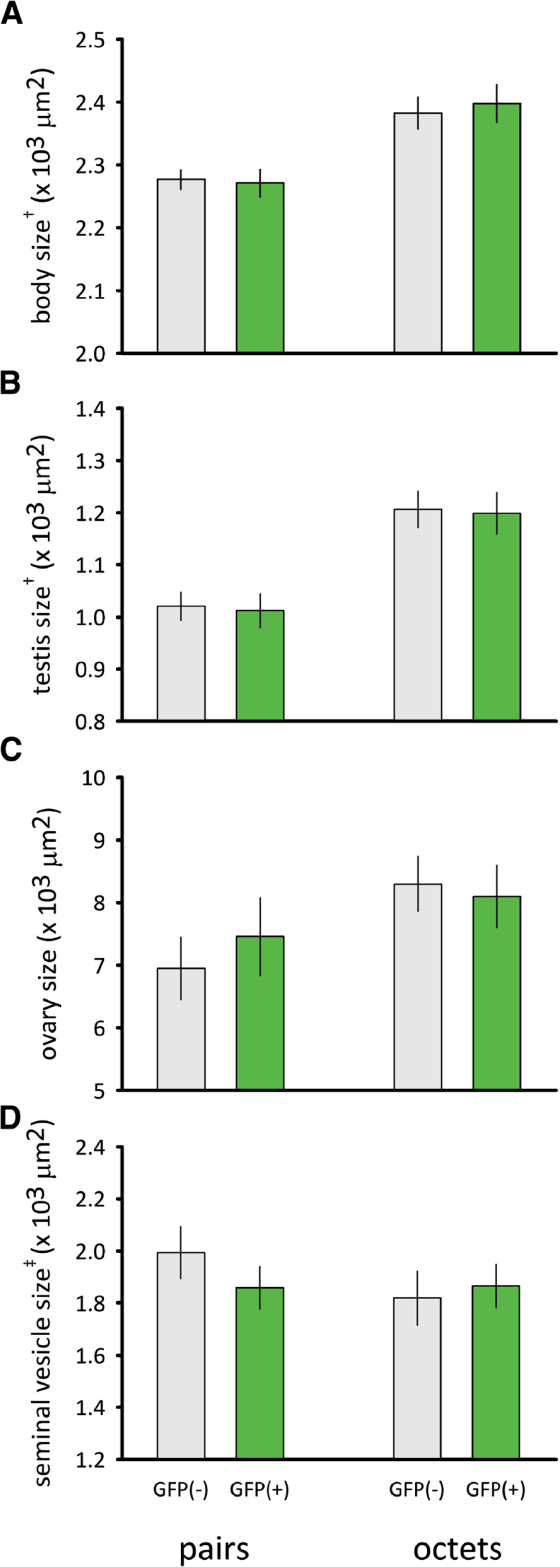
**Morphology of worms from the GFP(-) and GFP(+) lines.** Comparisons of body size **(A)**, testis size **(B)**, ovary size **(C)**, and seminal vesicle **(D)** between GFP(-) and GFP(+) individuals raised in groups of 2 (i.e. pairs), or 8 individuals (i.e. octets). We show means (±SE). † log transformation. ‡ square-root transformation. See text for statistics.

We found no difference in body size (*F*_*1,42*_ = 0.04, *P* = 0.85), testis size (*F*_*1,42*_ = 0.06, *P* = 0.81), ovary size (*F*_*1,42*_ = 0.10, *P* = 0.75) and seminal vesicle size (*F*_*1,40*_ = 0.29, *P* = 0.59) between GFP(+) and GFP(-) individuals (Figure 3). We did find the expected phenotypically plastic responses to the different social group sizes for most morphological traits (body size, *F*_*1,42*_ = 16.66, *P* < 0.001; testis size, *F*_*1,42*_ = 25.83, *P* < 0.001; ovary size, *F*_*1,42*_ = 3.29, *P* = 0.08; seminal vesicle size, *F*_*1,40*_ = 0.83, *P* = 0.37). Such plastic effects have previously been shown for outbred populations (e.g. [41,45]), and more recently also in the HUB1 line [43]. The line × group size interaction term was not significant for all traits, suggesting that GFP(+) show similar phenotypically plastic responses to groups size as the GFP(-) line (all P > 0.3).

### Experiment 2. Mating behaviour of GFP(+) vs. GFP(-) individuals

#### *Experimental setup*

To test if the individuals of the GFP(+) and GFP(-) differ in their mating rates, we performed mating trials of pairs. Specifically, we raised GFP(+) and GFP(-) individuals of two age cohorts in isolation and then performed mating trials, following the standard protocol described elsewhere [38]. We generated different crosses, namely GFP(+) × GFP(+), GFP(+) × GFP(-), and GFP(-) × GFP(-) in which the age cohorts were equally distributed over the treatments and the partners always belonged to the same age cohort. The mating behaviour was recorded for two hours, using the standard procedure described in [38], during which we counted the total number of matings performed while being blind with respect to the treatments.

#### *Statistics*

The sample sizes were 14, 11, and 14 for the GFP(+) × GFP(+), GFP(+) × GFP(-), and GFP(-) × GFP(-) crosses, respectively. To examine if the number of copulations differed between these 3 crosses, we fitted a generalized linear model (GLM), with a Poisson error distribution, a log-link function and a correction for overdispersion using the cross, the age cohort and the interaction cross × age cohort as fixed factors.

#### *Results*

**Figure 4:**
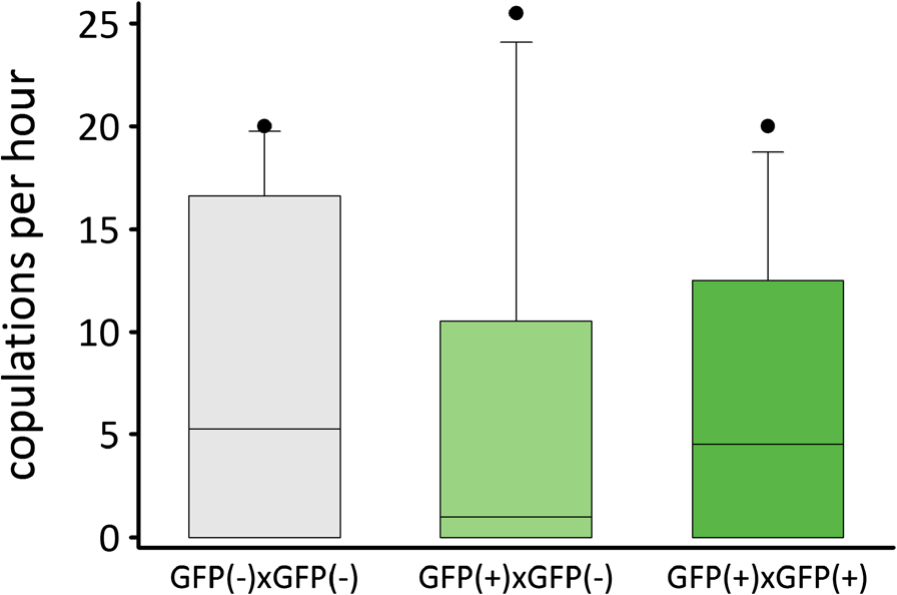
**Mating behaviour of worms from the GFP(-) and GFP(+) lines.** Comparisons of the mating rate of GFP(-) × GFP(-), GFP(+) × GFP(-), and GFP(+) × GFP(+) pairs. The boxes show the 25^th^ percentile, the median and the 75^th^ percentile. The whiskers show the 10^th^ and the 90^th^ percentile, and the dots show the outliers. See text for statistics.

We found that the 3 crosses had a similar copulation rate (χ^2^ = 2.36, *df* = 2, *P* = 0.31; Figure 4). Moreover, the younger cohort (1.1 ± 0.5, mean ± SE) copulated significantly less often than the older one (10.8 ± 1.6, mean ± SE; χ^2^ = 35.34, *df* = 1, *P* < 0.001), while the cross × age cohort interaction term was not significant (χ^2^ = 2.12, *df* = 2, *P* = 0.35).

### Experiment 3. Reproductive success of GFP(+) vs. GFP(-) individuals

#### *Experimental setup*

To test if the individuals from the GFP(+) and GFP(-) lines have similar male siring abilities and female reproductive success, we offered a partner to either GFP(+) or GFP(-) individuals, and compared the number of offspring they produced through their male and female sex functions. Specifically, we raised same-age GFP(+) and GFP(-) individuals in isolation. We formed pairs of GFP(+) × GFP(-) and GFP(-) × GFP(-) individuals for 3 days, which we then split to count the number of offspring each individual produced in isolation. We used the GFP(+) × GFP(-) pairs to estimate both the male siring ability of the GFP(+) worms, i.e. the number of offspring laid by its GFP(-) partner, and its female offspring production, i.e. the number of offspring the GFP(+) worm laid itself. In the GFP(-) × GFP(-) pairs, we randomly selected a priori one individual as a focal worm, for which we assessed the male siring ability and female offspring production as for the GFP(+) × GFP(-) pairs.

#### *Statistics*

The final sample sizes were 28 and 29 for the GFP(+) × GFP(-) and GFP(-) × GFP(-) treatments, respectively. We compared the male siring ability and the female offspring production of the GFP(+) and GFP(-) individuals by using Wilcoxon rank-sum tests.

#### *Results*

**Figure 5:**
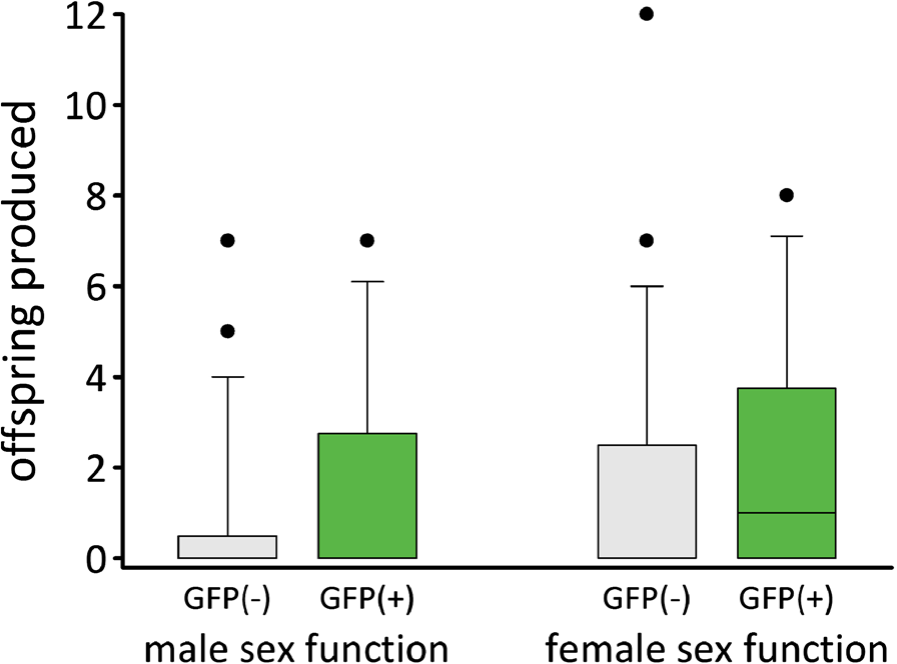
**Reproductive success of worms from the GFP(-) and GFP(+) lines.** Comparisons of the male siring ability and the female offspring production of the GFP(-) and the GFP(+) individuals. The boxes show the 25^th^ percentile, the median and the 75^th^ percentile. The whiskers show the 10^th^ and the 90^th^ percentile, and the dots show the outliers. See text for statistics.

The GFP(+) and GFP(-) lines did not differ in their male siring ability (*S* = 895, *N*_GFP(+)_ = 28, *N*_GFP(-)_ = 29, *P* = 0.12), or in their female offspring production (*S* = 883, *N*_GFP(+)_ = 28, *N*_GFP(-)_ = 29, *P* = 0.21) (Figure 5).

### Experiment 4. Worm phenotyping

#### *Experimental setup*

To determine if the GFP expression is a reliable phenotypic marker, we raised GFP(+) and GFP(-) individuals and assessed the GFP signal. For this, we raised 20 GFP(+) and 20 GFP(-) individuals and isolated them in wells of 60-well microtest plates (Greiner Bio-One, Germany). The GFP signal of the adult 40 worms was visually estimated by five different observers, while blind with respect to the treatment, using a binary scale as 0 (no GFP signal) and 1 (GFP signal).

#### *Results*

As expected, all GFP(+) and GFP(-) worms were scored correctly by all the 5 observers.

### Experiment 5. Sperm phenotyping - repeatability

#### *Experimental setup*

To test if we can reliably count the number of fluorescent sperm in the antrum of a GFP(-) recipient, we assessed the number of fluorescent sperm in the same recipient twice, and computed the repeatability of this measure. For this, we raised same-age individuals as follows: GFP(+) in pairs, GFP(-) in pairs, and GFP(-) in isolation. Note that the GFP(-) individuals used here were from an outbred line. After reaching maturity, we coloured the isolated GFP(-) individuals and formed groups of 3 individuals that were allowed to copulate for 100 min. These groups were composed of one coloured virgin GFP(-) individual used as a focal sperm recipient, one GFP(+) individual, and one GFP(-) individual. The virgin had thus two potential mating partners, a GFP(+) and a GFP(-) individual. Note that we grew the partners in pairs. We expect that these paired individuals copulated frequently, which promotes sperm production and avoids the accumulation of old sperm in the seminal vesicle of the sperm donor, potentially weakening GFP signal observed in sperm (see Experiment 7 below). We then isolated the focal sperm recipient from each group and estimated the number of total and fluorescent sperm received. Each sperm recipient was prepared and scored twice within a 30 min interval.

#### *Statistics*

The final sample size was 44 replicates. We assessed the repeatability between the first and the second count of both the total and the fluorescent sperm by computing intraclass-correlation coefficients [46]. We assessed the repeatability including and excluding recipients without sperm in storage (6 individuals for total sperm, 14 for fluorescent sperm), as they may lead to an overestimation of the repeatability.

#### *Results*

We found that counts of both the total number of sperm (r_i_ = 0.91, *F*_43,44_ = 22.0, *P* < 0.001) and the fluorescent sperm (r_i_ = 0.94, *F*_43,44_ = 31.1, *P* < 0.001) are highly repeatable, with slightly lower repeatabilities when excluding recipients that did not have sperm in storage (total sperm, r_i_ = 0.83, *F*_37,38_ = 10.5, *P* < 0.001; fluorescent sperm, r_i_ = 0.82, *F*_29,30 =_ 10.1, *P* < 0.001).

### Experiment 6. Sperm phenotyping - bright field vs. epifluorescence

#### *Experimental setup*

To test if we count the same number of fluorescent sperm under bright field and epifluorescence illumination, we counted sperm under both illumination techniques in recipients that had only received fluorescent sperm. Specifically, we raised same-age individuals in GFP(+) × GFP(-) pairs. We then assessed the number of received sperm in the GFP(-) recipient under both bright field and epifluorescence illumination. Hence, for each GFP(-) recipient, we obtained two sperm counts that are expected to match because all the received sperm must have been transferred by a GFP(+) donor.

#### *Statistics*

The final sample size was 57 replicates. We first tested if the number of sperm assessed under both illumination techniques correlate with each other, by using a Spearman’s correlation. We then tested whether we counted the same values in both illumination techniques by using the Wilcoxon signed-rank test. Individuals that did not receive sperm may bias these two tests, so we performed them with and without including worms having no sperm in storage (i.e. *N* = 12).

#### *Results*

**Figure 6:**
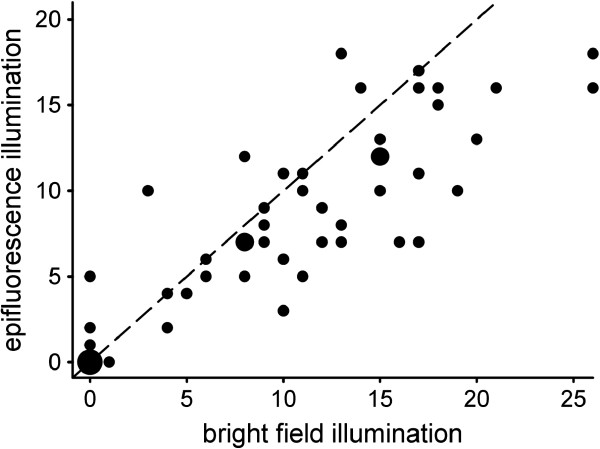
**Sperm phenotyping - bright field vs. epifluorescence.** Scatter plot of the number of fluorescent sperm assessed in a live GFP(-) recipient, under bright field and epifluorescence illumination. Small size dots represent individual replicates, intermediate size dots represent two overlapping replicates, and the big size dot represents 12 overlapping replicates. The dashed line displays the expected line of equality. See text for statistics.

Although the number of sperm assessed under bright field illumination was highly correlated with the corresponding number assessed under epifluorescence illumination (*r*_S_ = 0.88, *N* = 57, *P* < 0.001), we counted consistently more sperm under bright field illumination than under epifluorescence illumination (*S* = -262.5, *N* = 57, *P* < 0.001) (Figure 6). The same pattern was observed when excluding recipients that did not store sperm (Spearman’s correlation, *r*_S_ = 0.77, *N* = 45, *P* < 0.001; Wilcoxon signed-rank test, *S* = -262.5, *N* = 45, *P* < 0.001). The systematic discrepancy between the two illumination types may be due to an overestimation of the number of sperm under bright field illumination and/or an underestimation of the number of sperm under epifluorescence illumination (e.g. due to a loss of the GFP signal in some fluorescent sperm).

### Experiment 7. Sperm phenotyping - sperm ageing

#### *Experimental setup*

To investigate whether the GFP signal in the sperm is constantly emitted over time, we compared the strength of the GFP signal of presumably young and old sperm. For manipulating sperm age, we raised individuals either in groups or in isolation, thus controlling whether the produced sperm is constantly being spent for copulation or being accumulated in the seminal vesicle for several days. Specifically, we raised same-age individuals in 3 treatments, GFP(-) individuals in isolation (hereafter called control), GFP(+) individuals in isolation (hereafter called old sperm), and GFP(+) individuals in octets (hereafter called young sperm). Then, we prepared sperm cells for observation from the seminal vesicle of a donor, following a standard protocol described elsewhere [47], which allows us to assess the GFP signal of a single sperm cell. For each individual, we made movies of single sperm cell under epifluorescence illumination, and repeated this for 14.3 ± 0.2 sperm on average (±SE). Based on these movies, the strength of the GFP signal of each sperm was then scored using an ordinal scale with four categories: 0 (no GFP signal), 1 (weak GFP signal), 2 (intermediate GFP signal) and 3 (strong GFP signal).

#### *Statistics*

The sample size was 20 individuals per treatment. For each individual, we averaged the scores obtained from all scored sperm. As all controls were successfully scored as 0, we only compared the GFP signal of the old sperm and young sperm worms by using a *t*-test.

#### *Results*

**Figure 7:**
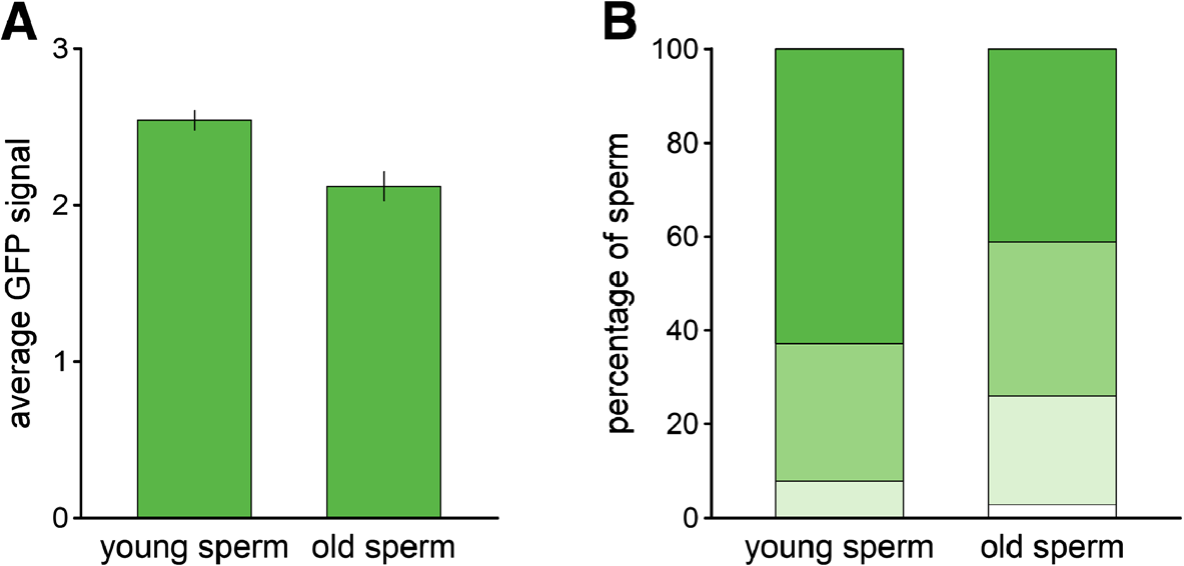
**Sperm phenotyping - sperm ageing. A**, barplots of the average (±1 SE) GFP signal observed in sperm of GFP(+) individuals carrying presumably young (from non-isolated donors) and old (from isolated donors) sperm. **B**, percentage of sperm scored as 0 (no GFP signal - white), 1 (weak GFP signal - light green), 2 (intermediate GFP signal - green) and 3 (strong GFP signal - dark green) of young and old sperm.

We found a clear age effect on the strength of the GFP signal, with older sperm having a lower GFP signal than the young sperm (*t*_38_ = -3.78, *P* < 0.001) (Figure 7A). In old sperm, we scored a larger percentage of sperm cells with lower GFP signal, as well as some sperm cells as having no GFP signal (3 ± 1%, mean ± SE) (Figure 7B). Hence sperm ageing might affect the strength of the GFP signal in sperm.

### Experiment 8. Mendelian segregation

#### *Experimental setup*

To test the inheritance patterns of the GFP marker, we assessed its segregation in offspring of crosses composed of known genotypes. First, we used pairs of GFP(+) × GFP(+), GFP(+) × GFP(-), and GFP(-) × GFP(-), to create offspring that were expected to be GFP(+) homozygous (hereafter *hom*^+^), heterozygous at the GFP locus (hereafter *het*), and GFP(-) homozygous (hereafter *hom*^*-*^), respectively. We then created pairs in five treatment groups: *hom*^+^ × *hom*^+^, *hom*^+^ × *hom*^*-*^, *het* × *het*, *het* × *hom*^*-*^, and *hom*^*-*^ × *hom*^*-*^, and subsequently assessed the GFP phenotype [i.e. GFP(+) or GFP(-)] of their offspring, which were collected over an extended time period. Assessment was made blind with respect to the treatment.

#### *Statistics*

The sample sizes (number of pairs) were 8 *hom*^+^ × *hom*^+^, 9 *hom*^+^ × *hom*^*-*^, 12 *het* × *het*, 11 *het* × *hom*^*-*^, and 13 *hom*^*-*^ × *hom*^*-*^. The offspring production was on average 35.9 ± 1.7 offspring (±SE) per pair. We tested if the GFP marker shows a Mendelian segregation assuming a dominant marker on a single diploid locus. For this, we reported when the proportion of GFP(+) offspring produced deviated from 1, 1 and 0 in the pairs of *hom*^+^ × *hom*^+^, *hom*^+^ × *hom*^*-*^, and *hom*^*-*^ × *hom*^*-*^ crosses, respectively. In addition, for each pair of the *het* × *het* and *het* × *hom*^*-*^ crosses, we statistically tested whether the observed frequencies of GFP(+) and GFP(-) offspring significantly deviated from the assumption of Mendelian segregation of 3:1 and 1:1 respectively, by using likelihood-ratio chi-square tests.

#### *Results*

The GFP marker follows an inheritance pattern in accordance with the presence of a dominant marker on a single diploid locus in most, but not all, of the pairs tested. Specifically, three *hom*^+^ × *hom*^*-*^ pairs produced 31:1, 38:1, and 6:7 GFP(+):GFP(-) offspring; one *het* × *het* produced 37:3 (χ^2^ = 8.30, *df* = 1, *P* = 0.004), and one *het* × *hom*^-^ pair 22:10 (χ^2^ = 4.62, *df* = 1, *P* = 0.032) GFP(+):GFP(-) offspring. The observed GFP(+):GFP(-) frequencies observed in the offspring of all the remaining pairs fit the expected values.

### Experiment 9. Sperm displacement

#### *Experimental setup*

We proved the potential of the GFP techniques to test whether the sperm stored in the antrum can be displaced by subsequent mating partners. For this we assessed the number of sperm from a GFP(+) donor in a GFP(-) recipient both before and after it was mated with a GFP(-) sperm competitor, and compared it with a control recipient that was not offered a second sperm donor. Specifically, we raised same-age individuals in pairs consisting of a GFP(+) sperm donor and a GFP(-) recipient. We isolated the sperm recipient, assessed the number of sperm received in its antrum (using bright field and epifluorescence illumination), and either kept it in isolation (i.e. called control treatment), or placed it with a second sperm donor, a coloured GFP(-) individual that had previously been in a GFP(-) × GFP(-) pair (i.e. called competition treatment). After one day, we sampled the recipient and assessed the number of total and fluorescent sperm received in its antrum a second time, with fluorescent sperm corresponding to the first donor’s sperm. The numbers of total and fluorescent sperm were assessed from movies recorded under bright field and epifluorescence illumination, respectively. We thus assessed the number of sperm received by the focal recipient from the first, GFP(+), donor both before and after the presence or absence of a GFP(-) sperm competitor.

#### *Statistics*

The final sample size was 13 replicates in the competition treatment, and 18 replicates in the control treatment. We statistically tested for sperm displacement by examining if the number of first donor sperm decreased more strongly in the competition treatment than in the control treatment. For this, we used a repeated-measures ANOVA with the number of sperm (i.e. either total sperm or first donor sperm) as the response variable, and time (i.e. first or second observation time-point), treatment (i.e. competition or control), and the interaction time × treatment as factors.

#### *Results*

**Figure 8:**
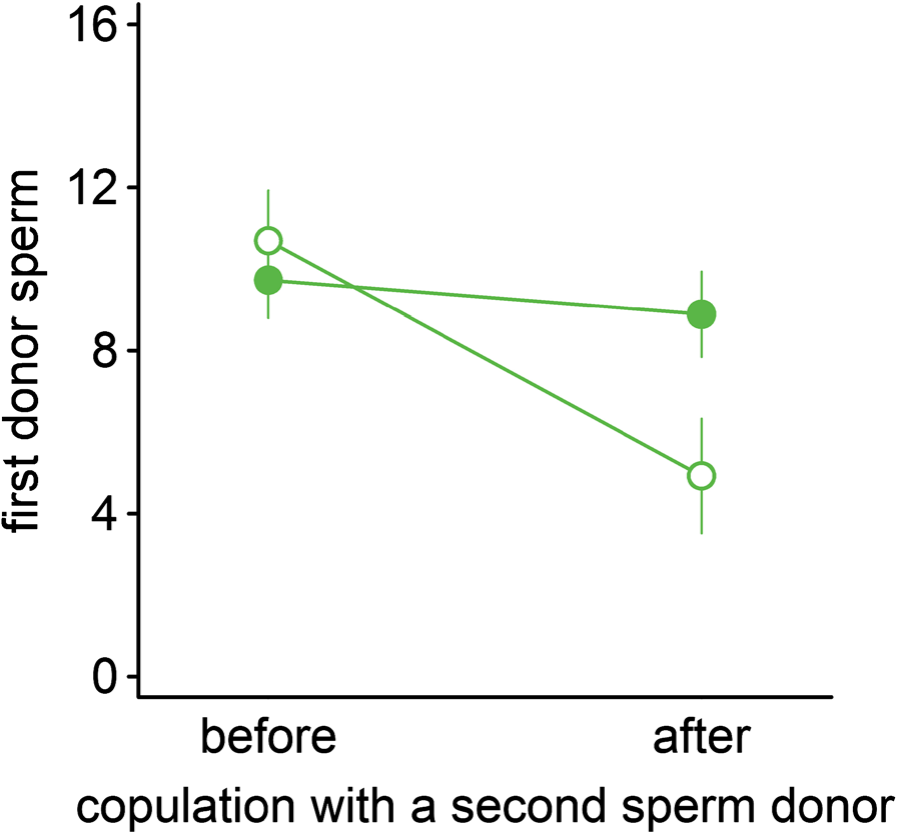
**Sperm displacement in *M. lignano*.** Means ± 1 SE of the number of first donor sperm, i.e. fluorescent sperm, stored in a GFP(-) individual before and after the presence of a second sperm donor (i.e. competition treatment - open circles), or twice without a second sperm donor (i.e. control treatment - filled circles). See text for statistics.

We found clear evidence for sperm displacement in *Macrostomum lignano* (Figure 8), as shown by a significant time × treatment interaction (*F*_1,29_ = 9.12, *N* = 31, *P* = 0.005), with the number of fluorescent sperm decreasing more in recipients that experienced a second sperm donor. We found a significant time effect (*F*_1,29_ = 16.32, *N* = 31, *P <* 0.001), and no overall treatment effect (*F*_1,29_ = 1.15, *N* = 31, *P* = 0.29).

The total number of sperm did not differ significantly between the two time-points (*F*_1,29_ = 0.05, *N* = 31, *P* = 0.83), and did not differ between the treatments (*F*_1,29_ = 1.22, *N* = 31, *P* = 0.28), but we found a significant effect of the interaction time × treatment (*F*_1,29_ = 6.36, *N* = 31, *P* = 0.018), with an increase of total sperm in recipients that experienced a second sperm donor.

## Discussion

The results suggest that the integration and the expression of the GFP construct in the HUB1 line of *M. lignano* do not affect any of the measured morphological and behavioural traits, as shown by the comparisons between GFP(+) and GFP(-) lines. Moreover, the GFP(+) line is a powerful and reliable tool for the study of sexual selection, as the GFP expression in sperm donors, their sperm and their sired offspring can easily be identified *in vivo*. The GFP marker seems to largely show Mendelian inheritance assuming a dominant allele on a single diploid locus, but further studies should determine if the few deviations found suggest another genetic model. Finally, we show a biological application of the GFP techniques by unambiguously demonstrating sperm displacement in *M. lignano*. In the following we discuss these main points in some more detail.

### Validation of the GFP marker: Reliability and limitations

Overall, the tests performed show that the GFP(+) line can be reliably used to study postcopulatory mechanisms of sexual selection. First, the GFP(+) line had similar morphological traits, mating rate and reproductive success than the GFP(-) line. Thus, the integration of the transgene as well as its expression does not seem to affect the performance of the GFP(+) worms. This was not necessarily expected, as the construct was presumably integrated in a random site in the genome, where it could have affected important gene functions. For instance, other GFP-expressing lines in *M. lignano* have shown a slower development than the wild type [36]. Similarly, the GFP and RFP (red fluorescent protein) expressing lines in *Drosophila melanogaster* have also been shown to suffer from detrimental fitness effects in male reproductive success [29]. Second, GFP expression provides a reliable tool for experimental reproductive biology: GFP(+) individuals are clearly and easily identifiable. Moreover, the high repeatability of fluorescent sperm counts in live recipients provides a powerful tool for studying sperm competition in real-time. Although we found a discrepancy in the number of received sperm under bright field and epifluorescence illumination, we show that this might partly be due to sperm ageing, as suggested by the small percentage of sperm without GFP signal in virgin GFP(+) worms. Potential problems arising from the underestimation of of GFP(+) can be easily circumvented with an appropriate experimental design that avoids the use of virgin or sexually isolated GFP(+) sperm donors as well as to preferentially use either only GFP(+) or only GFP(-) individuals as focal sperm donors.

The inheritance pattern of the GFP marker seems to largely follow Mendelian segregation assuming a dominant allele on a single diploid locus, as shown by the observed proportion of GFP(+) offspring from different crosses. However, in 5 pairs out of 53 we observed proportions that did not fit these expectations. Such rare events have repeatedly occurred also in large-scale experiments and in routine laboratory maintenance (Vizoso D.B., Marie-Orleach L., and Schärer L., unpublished observations). This could potentially be due to a phenotypic loss of expression (for instance due to silencing of the GFP marker or developmental problems), and/or due to a different genetic model (for instance having more than one GFP locus, or a biased segregation). The inheritance pattern of the GFP marker in the first few generations following transgene integration was also interpreted as a dominant allele on a single locus (see [36]). Nevertheless, Demircan [36] suggests that there could be multiple transgene integration sites, which are, however, expected to be located close to each other in the genome. Additional molecular analyses and large-scale multi-generational crosses are needed to better understand the genetic model driving the GFP expression.

In a series of preliminary tests (data not shown) we used qPCR on individual offspring of such unusual pairs to compare the amount of genomic DNA for two housekeeping genes, the promoter region and the region coding for the GFP protein. These analyses ruled out silencing as an explanation for the observed deviations, as all the GFP(-) offspring tested did not appear to carry the construct. Moreover, the dosages of the DNA construct in the GFP(+) worms were more variable than expected based on a single-locus diploid genetic model. These analyses were followed up by karyotyping of both the DV1 [our GFP(-)] and HUB1 [our GFP(+)] line, which identified a chromosomal polymorphism in these lines, namely two, three or four copies of the largest chromosome. If the insertion site of the GFP construct sits on this chromosome, this polymorphism would of course affect the dosage (Zadesenets K., personal communication; Schärer L., unpublished observation) and possibly account for the inheritance patterns observed. More detailed tests on this chromosomal polymorphism and the genomic location of the GFP construct are currently being performed.

An effective way of dealing with this issue is to experimentally assess the segregation patterns for each focal GFP(+) individual used in an experiment by pairing them with a virgin wild type individual and assessing the GFP status of the offspring in a large progeny array, either before or after an experiment. Individuals that show unexpected segregation patterns can then either be excluded from the experiment or their paternity estimates corrected based on the observed segregation patterns (Marie-Orleach L., Vizoso D.B. and Schärer L., unpublished observations).

### Sperm displacement

The unique opportunity to repeatedly quantify the contribution of a specific sperm donor to a pool of received sperm yielded new insights on the reproductive biology of *Macrostomum lignano*: individuals seem to displace sperm from previous donors and replace it with their own. Sperm displacement may be an underlying mechanism for last-male sperm precedence, i.e. the observation that the last sperm donor of a given recipient has a larger paternity share than expected under a fair raffle scenario [3,7,9]. In *M. lignano*, recent evidence shows that the second mating partner indeed sires more offspring than the first mating partner (Sandner P. and Schärer L., unpublished observations). The mechanism of sperm displacement in *M. lignano* is, at present, not known. We believe that some morphological traits of the copulatory organ and of the sperm may confer selective advantages in sperm offense and/or sperm defence (e.g. [48]). For instance, the shape of the male copulatory organ has been shown to be correlated with sperm-transfer success in this species [26]. Likewise, the complex shape of the sperm cells may have evolved as a consequence of sperm displacement, with stiff lateral bristles that can be involved in preventing the sperm from being removed from the antrum [49,50].

### Outlook

The availability of the GFP(+) line in *M. lignano* represents a new research tool that greatly improves the experimental possibilities in this emerging model organism. For instance, it has already been used to explore the relationship between mating group size and sex allocation [43]. By taking advantage of the GFP(+) line, Janicke et al. [43] were able to disentangle social group size (i.e. number of potential mating partners) from mating group size (i.e. number of actual mating partners) and then provide the most direct empirical support for a longstanding theoretical prediction of sex allocation theory [51,52]. Additionally, the GFP(+) line allows us to expose labelled individuals to different social or environmental conditions over an extended period of time, and to assess relevant traits, and their reaction norms, of a given individual or genotype. Furthermore, the GFP(+) line also allows us to disentangle episodes of postcopulatory sexual selection, namely selection on the number of sperm that enter the fertilising pool (i.e. sperm transfer success), and selection on fertilisation success of the transferred sperm (i.e. sperm fertilisation success). Therefore, we believe that the outlined method provides an approach towards a more quantitative and fine-scaled understanding of postcopulatory sexual selection.

## Conclusion

The experiments performed in this study show that the GFP(+) line in *Macrostomum lignano* is a reliable and powerful tool. This approach permits repeated non-invasive quantification of focal individuals during and after long-term interactions with GFP(-) individuals, such as the relative contribution of a sperm donor in a recipient *in vivo* and under competitive conditions, which was not possible before. We conclude that the availability of reliable GFP techniques in a transparent organism is a powerful tool, and represents a promising opportunity to reveal new insights in sexual selection as well as in other fields of biology.

## Availability of supporting data

The data sets supporting the results of this article are available in the Dryad repository, http://dx.doi.org/10.5061/dryad.tq43g[53].

## Competing interests

The authors declare that they have no competing interests.

## Authors’ contributions

LMO, TJ, DBV and LS designed the experiments. LMO, TJ and ME performed the experiments. LMO and LS analysed the data. DBV and LS established the DV1 line. LMO, TJ, DBV and LS wrote the manuscript. All authors read and approved the final manuscript.
